# Neonatal Calf Serum MAP Antibody Titre as a Potential Marker of Early-Life MAP Exposure

**DOI:** 10.3390/ani16131963

**Published:** 2026-06-25

**Authors:** Jonathan Hedgecock, Peter Plate, Steven van Winden

**Affiliations:** Pathobiology and Population Sciences, Royal Veterinary College, Hawkshead Lane, Hatfield AL9 7TA, UK; jhedgecock19@rvc.ac.uk (J.H.); pplate@rvc.ac.uk (P.P.)

**Keywords:** Johne’s disease, *Mycobacterium avium* subspecies *paratuberculosis*, calves, dairy cattle, colostrum, serum total protein

## Abstract

Johne’s disease (JD), caused by *Mycobacterium avium* subsp. *paratuberculosis* (MAP), is typically acquired early in life, yet detection of exposure during this period remains challenging. This study evaluated whether MAP-specific antibodies are passively transferred from dam to calf and detectable in neonatal calf serum, and whether these titres reflect early-life exposure to MAP. Neonatal calves (n = 38) from a commercial dairy herd were blood sampled within the first 10 days of life as part of routine assessment of passive transfer. Serum total protein (STP) was measured to assess colostrum intake, and residual serum was analysed for MAP antibody titres using an indirect ELISA. Multivariable linear regression was used to evaluate associations between calf MAP antibody titres, STP, and dam JD serological status. Calf serum MAP antibody titres were positively associated with STP (*p* < 0.001) and differed according to dam serological status, with higher titres observed in calves born to MAP-seropositive dams (*p* = 0.025). A significant interaction between STP and dam status indicated that the relationship between passive transfer efficiency and MAP antibody levels varied by dam infection status. These findings support the biological plausibility of passive transfer of MAP-specific antibodies via colostrum and suggest that neonatal calf serology may reflect exposure to an early-life epidemiological risk period associated with maternal MAP exposure and the calving environment rather than infection status within the calf itself. Given the modest sample size and single-herd design, these findings should be interpreted as exploratory and hypothesis-generating.

## Simple Summary

Johne’s disease is a chronic infectious disease of cattle caused by *Mycobacterium avium* subsp. *paratuberculosis* (MAP). Infection usually occurs early in life, although clinical signs may not develop for several years. Detecting exposure during this early period remains challenging. In this study, calves less than 10 days old were blood sampled for passive transfer testing and then also tested for MAP specific antibodies. The results showed that calves born to MAP-seropositive dams had higher neonatal antibody levels than calves born to MAP-seronegative dams. In addition, the relationship between antibody levels and serum total protein concentration, a marker of colostrum absorption, differed according to maternal MAP serostatus. These findings are consistent with passive transfer of MAP-specific antibodies through colostrum and suggest that neonatal antibody measurements may provide information about early-life MAP exposure during the calving period. While further validation across multiple herds is required, neonatal serology may offer a complementary approach for investigating early-life MAP exposure alongside existing Johne’s disease control strategies.

## 1. Introduction

Johne’s disease (JD), or paratuberculosis, is a chronic granulomatous enteritis of ruminants caused by *Mycobacterium avium* subspecies *paratuberculosis* (MAP), an acid-fast, obligate intracellular pathogen with marked environmental persistence [1,2]. The disease is of particular importance in dairy cattle, where infection typically occurs early in life, but clinical signs may not manifest for several years, if at all. When present, clinical disease is characterised by progressive weight loss, diarrhoea, declining productivity and eventual death, usually following an incubation period of two to seven years [3]. This prolonged subclinical phase complicates detection and control, allowing infected animals to remain within the herd for extended periods while contributing to onward transmission.

JD imposes substantial economic costs on the dairy industry through reduced milk yield, impaired reproductive performance, increased mastitis risk and premature culling, making control both a welfare and financial priority [4,5]. In the United Kingdom, where herd-level prevalence remains high, JD also attracts ongoing public and veterinary public health interest due to its debated association with Crohn’s disease in humans, although a direct causal link has not been conclusively demonstrated [1,6].

Transmission of MAP occurs predominantly via the faecal-oral route, with calves exhibiting heightened susceptibility during the first weeks to months of life. This age-related vulnerability is likely multifactorial, reflecting a combination of biological and management-related factors. Neonatal adaptations of the gastrointestinal tract that facilitate the absorption of colostral immunoglobulins, including the activity of M cells within Peyer’s patches, may also permit translocation of MAP across the intestinal epithelium [7,8,9]. In addition, the naïve immune status of the neonatal calf and early-life management practices increase the likelihood of exposure to high infectious doses, particularly in herds with moderate to high prevalence. Vertical transmission has also been documented, with MAP detected in approximately 40% of foetuses from clinically affected cows, further emphasising the importance of the dam–calf interface in JD epidemiology [10].

Following infection, JD typically progresses through a prolonged subclinical phase characterised by a predominantly cell-mediated immune response that restricts bacterial proliferation within the intestinal mucosa and associated lymphoid tissue through granuloma formation [11]. As disease advances, a shift towards a humoral immune response occurs, coinciding with increased bacterial burden, faecal shedding and, eventually, clinical disease [12,13]. This immunological transition underpins many of the diagnostic challenges associated with JD. Antibody-based tests, while widely used in surveillance programmes, have limited sensitivity in early and subclinical infection, resulting in delayed detection of infected animals that may already be shedding MAP into the environment [14,15].

Routine JD control in UK dairy herds therefore relies largely on repeated milk or serum antibody ELISA testing combined with management interventions and test-and-cull strategies. Animals identified as high-risk based on sustained or rising antibody titres are frequently classified operationally as “red cows” and managed accordingly, often through targeted culling or restricted breeding [16]. While these approaches can reduce prevalence over time, they are inherently limited by the poor sensitivity of antibody tests during the period when transmission risk may be greatest. Faecal culture and PCR-based methods offer improved sensitivity and specificity, but their cost, labour intensity and delayed turnaround times restrict their use in routine herd-wide surveillance [17]. Emerging technologies such as bacteriophage-based assays, including Actiphage^®^, provide more rapid detection of viable MAP and have shown promise as adjunct diagnostic tools [18,19], but are primarily used for confirmation rather than routine screening in most control programmes.

Recent work has therefore emphasised the value of integrating repeated test results with animal-level and herd-level information to improve risk estimation. Bayesian and probabilistic modelling approaches that incorporate serial antibody measurements, herd prevalence and dam infection status have been shown to provide a more nuanced, continuous estimate of infection probability than categorical test interpretations alone [20]. Such approaches have the potential to support more informed management decisions, particularly with respect to identifying animals at increased risk of vertical transmission or environmental shedding. However, even these models remain constrained by the biological limitations of antibody production in early infection.

Passive transfer of immunity in cattle occurs almost entirely through ingestion and intestinal absorption of colostral immunoglobulins during the first hours of life. Unlike many mammalian species, the bovine placenta does not permit significant transplacental transfer of immunoglobulins, and calves are therefore born essentially agammaglobulinaemic [21]. Colostrum contains extremely high concentrations of immunoglobulin G (IgG), typically ranging between 50 and 100 g/L, alongside lower concentrations of IgM and IgA, and provides both systemic and mucosal immune protection during the early neonatal period. Absorption occurs through specialised enterocytes in the small intestine capable of non-selective pinocytosis of intact immunoglobulin molecules, a process that declines rapidly after birth as intestinal “closure” occurs, typically within 24 h. As a consequence, circulating antibody concentrations in the neonatal calf closely reflect both the immunoglobulin concentration of the ingested colostrum and the efficiency with which these antibodies are absorbed into the systemic circulation.

Colostrum also contains pathogen-specific antibodies reflecting the antigenic exposure history of the dam. These antibodies are generated as part of the maternal adaptive immune response and are selectively concentrated in the mammary gland during late gestation through active transport mechanisms mediated by Fc receptors. As a result, calves that successfully ingest adequate volumes of high-quality colostrum acquire circulating antibodies against pathogens present within the dam’s immunological repertoire. In the context of Johne’s disease, this raises the possibility that MAP-specific antibodies produced during maternal infection may also be transferred to the calf via colostrum. If present in sufficient concentration, such antibodies could be detected in neonatal calf serum using conventional antibody assays, providing a potential indirect marker of maternal exposure and early-life environmental risk.

Therefore, an under-explored but biologically plausible opportunity to improve early risk assessment lies in the evaluation of passive transfer of MAP-specific antibodies from dam to calf. As colostrum contains substantially higher concentrations of pathogen-specific immunoglobulins than milk or serum, and calves are agammaglobulinaemic at birth, post-colostral serum antibody levels have the potential to be used as a proxy for maternal exposure [22]. Longitudinal studies have demonstrated that calves born to dams that seroconvert up to 12 months after calving have an increased risk of developing JD, suggesting that maternal infection may precede detectable antibody responses and that calves may be exposed during this pre-seroconversion period [23]. Whether MAP-specific antibodies are passively transferred in sufficient quantity to be detected in calf serum, and whether such transfer reflects dam infection status, remains unclear.

Understanding the extent and determinants of passive transfer of MAP-specific antibodies could offer a novel, earlier indicator of dam infection and calf exposure, with practical implications for JD control. If calf serum antibody levels reflect subclinical maternal infection, this information could be used to inform earlier culling or breeding decisions, refine replacement heifer selection, and reduce the risk of vertical transmission before dams themselves test positive in routine surveillance.

The overall aim of the present study was therefore to evaluate the passive transfer of MAP-specific antibodies from dam to calf. Specifically, the study sought to determine: (i) whether MAP-specific antibodies are detectable in calf serum following colostrum intake; (ii) whether the magnitude of this transfer is associated with the overall efficacy of passive immunity transfer, as measured by calf serum total protein concentration; and (iii) whether MAP-specific antibody levels in calves are associated with the JD infection status of their dams, defined by seropositivity for MAP antibodies.

By addressing these questions, this study aims to establish the patency of MAP-specific passive antibody transfer from dam to calf and to evaluate whether calf serum antibody levels can be used as an indirect marker of early-life exposure during a quantifiable high-risk period, reflecting both contact with a MAP-positive dam and the environmental conditions in which transmission is most likely to occur.

## 2. Materials and Methods

### 2.1. Study Population and Ethical Approval

Ethical approval for this study was granted by the Clinical Research and Ethical Review Board at the Royal Veterinary College, London, UK (URN 2024 2297-2). Written consent for use of residual serum samples was obtained from the participating farm in accordance with this approval.

The study population comprised 38 dairy calves born between February and August 2025 on a single organic spring and autumn block calving Holstein-Friesian dairy farm in South-West England. The farm participated in routine JD surveillance through quarterly milk antibody ELISA testing conducted via National Milk Records (NMR), Chippenham, UK, with laboratory testing accredited to ISO 17025 [24]. Calves were routinely blood sampled within the first 10 days of life (median: 3 days; interquartile range: 2–5 days) for serum total protein (STP) measurement as part of standard colostrum management monitoring, and residual serum was retained for subsequent analysis of MAP-specific antibody titres.

Calf and colostrum management on the study farm is as follows: (i) irrespective of JD status dams calve in the same straw dry cow yard; (ii) dam and calf pairs separated and placed into calving pens following/during parturition for 12–24 hrs whilst the calf is allowed to suckle colostrum; (iii) following this calves are moved into individual pens and the dams are placed into the main milking group; (iv) calves are only occasionally supplemented with colostrum if weak or sick; (v) calves are then fed whole milk for 3 days before switching to milk replacer.

The study herd was known to have ongoing MAP exposure based on routine quarterly JD surveillance, including the presence of repeatedly MAP-seropositive adult cattle within the milking herd during the study period.

### 2.2. Definition of Dam Johne’s Disease Status

Calves were classified according to the JD status of their dam at the time of calving. A dam was defined as MAP antibody positive if a single quarterly milk ELISA result exceeded an S/P% of 30 at any point prior to the calving event. Animals that had no positive milk ELISA results throughout their recorded testing history were classified as negative. Based on these definitions, calves were allocated into two groups: calves born to MAP seropositive dams and calves born to MAP seronegative dams.

Because milk ELISA testing was performed quarterly as part of routine herd surveillance, the interval between the most recent dam ELISA result and calving varied between animals. Exploratory analyses evaluating the relationship between dam milk ELISA values and the interval between testing and calving demonstrated temporal variability in measured antibody titres among seropositive animals. Consequently, dam status was retained as a biologically pragmatic categorical exposure variable rather than modelling historical dam MAP ELISA values directly as continuous predictors of neonatal calf antibody titres.

### 2.3. Blood Sampling and Serum Handling

Blood samples were collected by jugular venepuncture into plain vacutainer tubes in accordance with the farm’s routine procedures for STP assessment. Following clot formation, samples were centrifuged, STP was measured using a calibrated optical refractometer (unbranded, but calibrated and validated against accredited laboratory), and residual serum was aliquoted into labelled Eppendorf tubes and stored frozen until submission for laboratory analysis. Calf age at blood sampling was recorded in days and evaluated in preliminary analyses as a potential explanatory variable.

### 2.4. Serum Total Protein Measurement

Serum total protein concentration was measured in grams per litre (g/L) using a calibrated optical refractometer (unbranded, but calibrated and validated against accredited laboratory) as an indirect assessment of colostrum intake and efficacy of passive transfer. Calves were categorised according to Agriculture and Horticulture Development Board (AHDB) reference thresholds as having good (>55 g/L), marginal (50–55 g/L), or poor (<50 g/L) passive transfer [25]. In the analysis, all calves were used within the primary statistical models, with STP treated as a continuous explanatory variable representing passive transfer efficiency across the observed biological range. Sensitivity analyses restricted to calves with STP > 55 g/L were additionally performed to evaluate the robustness of observed associations.

### 2.5. Measurement of MAP-Specific Antibody Titres

Residual serum samples were submitted to National Milk Records Ltd., Wolverhampton, UK, for measurement of MAP-specific antibody titres using an indirect ELISA. Results were expressed as sample-to-positive (S/P) percentage (%) values, calculated as the ratio of sample optical density relative to that of a positive control. Although interpretative thresholds for this assay are validated for adult cattle, no established diagnostic thresholds exist for neonatal calves. Consequently, neonatal calf S/P% values were interpreted as continuous quantitative measurements of passively derived antibody signal rather than as diagnostic indicators of calf infection status.

### 2.6. Statistical Analysis

Statistical analyses were performed using IBM SPSS Statistics (Version 29; IBM Corp., Armonk, NY, USA), with statistical significance defined as *p* < 0.05. The outcome variable of interest, the neonatal calf serum MAP antibody S/P%, was approximately normally distributed (Kolmogorov–Smirnov test: *p* > 0.05), supporting the use of parametric methods.

A multivariable fixed-effects linear regression model was constructed to evaluate associations between neonatal calf serum MAP antibody S/P% values and explanatory variables. Variables included within the initial model were selected a priori based on biological plausibility and mechanistic relevance to passive antibody transfer. These included STP concentration, dam MAP serostatus, calf age at sampling, and the interaction between STP and dam serostatus.

Additional exploratory variables, including calf sex, breed, and dam parity, were evaluated in preliminary analyses but were not retained within the final model due to a lack of statistical support and the need to minimise overfitting given the modest sample size. Regression coefficients, standard errors, 95% confidence intervals, and associated *p*-values were reported for final model estimates.

Prior to model construction, scatterplots and residual diagnostics were examined to assess the linearity of relationships between explanatory variables and the outcome variable. Homoscedasticity and normal distribution of residuals were evaluated through visual inspection of residual plots and quantile–quantile plots. No substantial deviations from model assumptions were identified.

The interaction between STP concentration and dam MAP serostatus was specified a priori on biological grounds. Passive transfer efficiency determines the quantity of immunoglobulins absorbed by the calf, while dam serostatus determines whether MAP-specific antibodies are present within the maternal immunoglobulin pool. The interaction term therefore allowed assessment of whether increasing passive transfer efficiency was associated with greater neonatal MAP antibody titres specifically in calves born to MAP-seropositive dams.

## 3. Results

In total, 38 calves were included in the initial sample frame. The median STP concentration was 69 g/L (IQR: 58–78 g/L), and a median MAP S/P% of 23.4 (IQR: 13.4–37.4). Five calves were considered to have an inadequate passive transfer, defined as a STP concentration ≤ 55 g/L. Calf age at sampling was not significantly associated with neonatal MAP S/P% values in preliminary analyses and was therefore not retained within the final multivariable model.

In multivariable linear regression analysis, a significant interaction was identified between STP concentration and dam MAP serostatus (interaction coefficient: −14.3; 95% confidence interval [CI]: −25.2 to −3.3; *p* = 0.011), indicating that the relationship between passive transfer efficiency and neonatal calf MAP antibody titres differed according to maternal serostatus. Specifically, increasing STP concentration was associated with higher neonatal calf MAP S/P% values among calves born to MAP-seropositive dams (STP coefficient: 19.5; 95% CI: 10.1 to 28.9; *p* < 0.001), whereas this relationship was substantially attenuated among calves born to MAP-seronegative dams.

**Figure 1:**
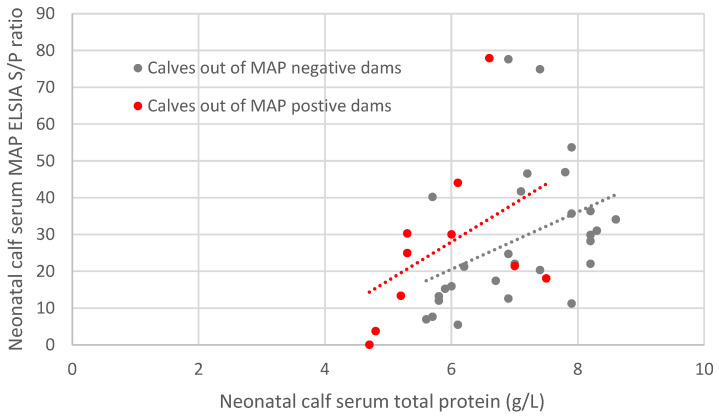
Relationship between neonatal calf serum MAP ELISA (S/P%) and STP concentration, stratified according to dam MAP serostatus. Points represent individual neonatal calves and dashed lines show fitted regression relationships. The divergence of slopes indicates that the relationship between passive transfer efficiency and neonatal calf MAP antibody titres differed according to maternal MAP serostatus (interaction *p* = 0.011).

Figure 1 illustrates the relationship between neonatal calf MAP S/P% and STP concentration stratified by dam MAP serostatus. The divergence of the fitted regression lines reflects the significant interaction between passive transfer efficiency and maternal serostatus.

Sensitivity analyses restricted to calves with STP > 55 g/L yielded materially similar findings, including persistence of the interaction between STP concentration and dam MAP serostatus (*p* = 0.040).

Given the limited sample size, results should be interpreted as exploratory.

## 4. Discussion

This study provides evidence that MAP-specific antibodies are detectable in the serum of neonatal calves and that their presence is jointly determined by the efficiency of passive transfer and the MAP serostatus of the dam. The observed association between calf serum MAP antibody S/P%, STP, and dam JD status supports the biological plausibility of maternally derived MAP-specific antibody transfer via colostrum. Importantly, the significant interaction between STP and dam status indicates that increasing passive transfer is associated with higher MAP antibody titres only when the dam is seropositive, consistent with a biologically constrained and specific transfer pathway.

Unlike studies that have investigated MAP antibody ELISA responses in older calves or youngstock as markers of early infection [26], this study focuses explicitly on the neonatal period and the patency of passive transfer. Detection of MAP-specific antibodies in calves within the first days of life cannot reasonably be interpreted as evidence of infection but rather reflects exposure to antibodies derived from colostrum. The observed interaction between maternal serostatus and STP concentration is consistent with this interpretation, as increasing passive transfer efficiency was associated with increasing neonatal MAP antibody titres primarily among calves born to MAP-seropositive dams.

A further point requiring clarification is the interpretation of MAP antibody ELISA results in neonatal calves. Antibody-based assays are generally considered to have limited sensitivity for detecting MAP infection in young animals because the humoral immune response develops only after prolonged intracellular infection and progression toward the multibacillary stage of disease. For this reason, ELISA testing of calves or youngstock is not recommended as a diagnostic method for identifying infected animals within JD control programmes. However, the objective of the present study differs fundamentally from that diagnostic context. In neonatal calves sampled within the first days of life, detectable antibodies cannot plausibly arise from an endogenous immune response because insufficient time has elapsed for antigen processing, clonal expansion and antibody production. Instead, circulating antibodies present during this period are overwhelmingly derived from passive transfer of maternal immunoglobulins absorbed from colostrum.

This distinction is important because it fundamentally changes the biological interpretation of ELISA measurements in the neonatal period. Rather than representing evidence of infection in the calf, detectable MAP antibody titres represent the presence of maternally derived immunoglobulins reflecting the dam’s antigen exposure history. The ELISA assay therefore functions in this context as a quantitative measurement tool rather than a diagnostic classifier. Indeed, the very limitations that restrict the assay’s diagnostic sensitivity for detecting early infection in calves become largely irrelevant when the measurement target is passive antibody transfer. When interpreted in this manner, neonatal ELISA titres provide information about maternal immunological status and the antigenic environment surrounding the calving event rather than the infection status of the calf itself.

A similar interpretative framework has been applied in other infectious disease systems where maternally derived antibodies are used as markers of early-life exposure risk or maternal immune status. A comparable interpretative framework has been used in bovine neosporosis, where serological relationships between dams and offspring have been used to investigate vertical transmission dynamics. Studies have demonstrated that calves born to seropositive dams are substantially more likely to exhibit detectable antibodies at birth or shortly thereafter, reflecting maternal infection status and in utero exposure risk [27,28]. In livestock epidemiology, measurement of passive antibodies in neonatal animals has been used to infer maternal vaccination status, pathogen circulation within herds, and the timing of exposure events within production systems. Within the context of Johne’s disease, neonatal antibody measurements may therefore offer a complementary approach to understanding the epidemiological conditions present at the time of birth, particularly in situations where maternal infection is present but has not yet been detected through routine surveillance testing.

The interaction observed between STP and dam JD status is particularly informative. While STP is a general indicator of immunoglobulin absorption, its relationship with MAP-specific antibody levels differed markedly depending on maternal serostatus. This divergence supports the specificity of the signal and argues against non-specific binding or assay artefact as the primary driver of the observed associations. These findings align with previous work demonstrating that colostral antibody concentrations are directly reflected in neonatal serum immunoglobulin levels [29], while extending this principle to MAP-specific antibodies in the context of JD control.

From a JD epidemiological perspective, the relevance of this finding lies not in diagnostic classification, but in risk approximation. Calves with detectable MAP-specific antibodies are consistent with exposure to a dam with MAP antibodies and therefore been present in an environment with active MAP circulation at calving. This provides a temporarily precise marker of exposure to a quantifiable risk period, encompassing both potential colostral and faecal transmission routes. Such an approach complements existing JD control frameworks that rely heavily on dam testing histories, which may be limited by the low sensitivity of MAP serology and the temporal lag between infection and seroconversion [23,30].

From a herd-level disease control perspective, this finding may have practical implications for the interpretation of early-life exposure risk. Current JD control programmes rely heavily on retrospective identification of infected dams based on serial milk or serum ELISA testing. However, because antibody responses often arise relatively late in the course of infection, calves may be born to dams that are infected but not yet detectable through routine surveillance. Detection of MAP-specific antibodies in neonatal calves could therefore act as an indirect indicator of undetected maternal infection or heightened environmental contamination at the time of calving. When interpreted alongside existing herd surveillance data, such information may assist herd managers and veterinarians in identifying cohorts of replacement heifers that experienced elevated early-life exposure.

In practical terms, neonatal serological measurements could potentially complement existing risk-based management strategies already employed within JD control schemes. The value of neonatal serology therefore lies primarily in providing additional contextual information about the epidemiological environment in which calves were born rather than serving as a definitive diagnostic test.

In exploratory analyses conducted for hypothesis generation, neonatal calf serum total protein concentration and MAP antibody S/P% showed associations with subsequent adverse dam outcomes, including post-calving seroconversion or culling. However, given the limited sample size, composite nature of these outcomes, and post hoc design of these analyses, these observations were not explored further and should be interpreted with caution.

Several limitations must be acknowledged. The sample size was modest, and the study was conducted on a single farm with specific husbandry practices, including prolonged dam–calf contact. As such, the results should be interpreted as exploratory and hypothesis-generating. Additionally, dam JD status was defined using historical milk ELISA results, which may misclassify some infected animals. However, such misclassification would be expected to bias associations toward the null hypothesis, suggesting that the observed effects are conservative estimates. Furthermore, due to the nature of separating calving dams from the calving group based on visual inspection of parturition, it may have been possible for calves to be born and cross suckle colostrum from another dam before they were placed into a maternity pen. This effect was likely not frequent and if present would also be expected to bias associations towards a null hypothesis, further suggesting that these observed effects are conservative estimates.

Several additional methodological considerations warrant discussion. First, measurement of MAP-specific antibodies was performed using an ELISA assay validated for use in adult cattle. While the assay reliably quantifies relative antibody concentrations, interpretative thresholds for neonatal calves have not been established. Consequently, antibody titres were analysed as continuous variables rather than categorised as positive or negative. This approach avoids inappropriate diagnostic interpretation but also limits direct comparison with adult testing frameworks used in herd surveillance programmes.

Second, colostrum quality and intake volume were not directly measured in this study. Serum total protein concentration provides a well-validated proxy for successful passive transfer and reflects the combined effects of colostrum immunoglobulin concentration, intake volume and intestinal absorption efficiency. Nevertheless, variation in colostral antibody concentration between dams could influence the magnitude of MAP antibody transfer independent of calf absorption efficiency. Future studies incorporating direct measurement of colostrum immunoglobulin and pathogen-specific antibody concentrations would allow more precise quantification of these relationships.

Third, no other MAP infection testing, such as faecal PCR or culture, was performed in either dams or calves. Given the early neonatal sampling period and prolonged incubation dynamics of MAP infection, direct pathogen detection in calf faeces would be unlikely to provide biologically interpretable information regarding early infection status. The present study was therefore designed to evaluate serological associations consistent with passive antibody transfer rather than to determine infection status directly and it could serve as dam-calf proximity measure, potentially linked with MAP transfer.

Finally, the cross-sectional design of the present study precludes assessment of how neonatal MAP antibody titres change over time or whether they are associated with subsequent infection outcomes. Maternal antibodies decline progressively during the first weeks to months of life as passively acquired immunoglobulins are metabolised. Longitudinal sampling would therefore be required to characterise the temporal dynamics of MAP-specific antibody decay and to determine whether early antibody exposure has any predictive relationship with later JD infection status.

Future research should therefore focus on larger multi-herd longitudinal studies that integrate neonatal serology with detailed dam testing histories, environmental sampling and long-term follow-up of calf health outcomes. Such work would allow evaluation of whether neonatal MAP antibody titres correlate with subsequent infection risk, disease progression or shedding dynamics. In addition, investigation of colostral MAP antibody concentrations and their relationship to maternal disease stage could further clarify the biological mechanisms underlying passive antibody transfer in JD. Integration of these data with probabilistic JD risk models may ultimately improve early-life risk stratification and inform more targeted management strategies aimed at interrupting transmission during the most vulnerable stages of the production cycle.

## 5. Conclusions

This study provides evidence that MAP-specific antibodies are detectable in neonatal calf serum during the first days of life and that neonatal antibody titres are associated with both serum total protein concentration and maternal MAP serostatus. The observed interaction between serum total protein and dam status indicates that the relationship between passive transfer efficiency and dam serostatus supports the biological plausibility of maternally derived MAP-specific antibody transfer via colostrum.

These findings suggest that neonatal calf MAP antibody titres may reflect exposure to a defined early-life epidemiological risk period associated with maternal MAP exposure and the calving environment, rather than indicating infection status within the calf itself. In this context, neonatal serology may offer a biologically grounded adjunct measure that could complement existing Johne’s disease surveillance and risk assessment approaches when interpreted alongside herd-level information.

However, given the modest sample size, single-herd design and absence of direct colostrum or maternal serum measurements at calving, the findings should be interpreted cautiously and considered hypothesis-generating. Further longitudinal and multi-farm studies incorporating direct colostrum measurements and long-term calf follow-up are required to determine the epidemiological and practical significance of neonatal MAP antibody titres within Johne’s disease control programmes.

## Data Availability

Dataset available on request from the authors.

## Abbreviations

The following abbreviations are used in this manuscript:

JD	Johne’s Disease
MAP	*Mycobacterium avium* subsp. *paratuberculosis*
STP	Serum Total Protein
ELISA	Enzyme Linked Immunosorbent Assay
PCR	Polymerase Chaine Reaction
NMR	National Milk Records
AHDB	Agriculture and Horticulture Development Board
S/P%	Sample-to-Positive Percentage
